# In Defense of Post Hoc Explanations in Medical AI

**DOI:** 10.1002/hast.4971

**Published:** 2026-02-04

**Authors:** Joshua Hatherley, Lauritz Aastrup Munch, Jens Christian Bjerring

**Keywords:** artificial intelligence, explainability, clinical decision‐making, ethics, post hoc explanations, bioethics

## Abstract

Since the early days of the explainable artificial intelligence movement, post hoc explanations have been praised for their potential to improve user understanding, promote trust, and reduce patient‐safety risks in black box medical AI systems. Recently, however, critics have argued that the benefits of post hoc explanations are greatly exaggerated since they merely approximate, rather than replicate, the actual reasoning processes that black box systems take to arrive at their outputs. In this paper, we aim to defend the value of post hoc explanations against this recent critique. We argue that even if post hoc explanations do not replicate the exact reasoning processes of black box systems, they can still improve users’ functional understanding of black box systems, increase the accuracy of clinician‐AI teams, and assist clinicians in justifying their AI‐informed decisions. While post hoc explanations are not a silver‐bullet solution to the black box problem in medical AI, they remain a useful strategy for addressing it.

## Article

Medical artificial intelligence has the potential to improve patient health and safety, reduce inefficiencies in medical decision‐making, strengthen clinician‐patient relationships, and increase the accessibility of high‐quality medical care.^1^ Increasingly, these systems are making their way into clinical practice. Indeed, the rate of regulatory approvals for artificial intelligence (AI) systems designed to assist in all manner of clinical tasks—including diagnosis, risk prediction, and surgical decision‐making—have rapidly accelerated over the past five to ten years.^2^ While clinical adoption of medical AI systems remains nascent, clinical usage of these systems has grown exponentially since 2018.^3^ Recent developments in generative AI and foundation models appear only to be escalating these trends.

A long‐standing concern with AI systems in high‐stakes decision‐making environments is that their inner workings are opaque.^4^ For deep neural networks and the like, not even the systems’ designers can scrutinize the reasoning behind each of these systems’ outputs. In medicine, this has prompted a range of concerns relating to patient safety, trust, health equity, and patient autonomy, among other things.^5^ In some cases, experts have gone so far as to recommend that “black box” AI systems be banned from use in high‐stakes decision‐making environments entirely.^6^

“Post hoc explanation models” refers to a suite of AI systems designed to reverse engineer explanations for the outputs and operations of black box systems on the basis of their prior record of inputs and outputs. These explanations have been praised for their potential to address or, indeed, overcome the black box problem by rendering these systems “interpretable” or “explainable.”^7^ Several critics have recently argued that post hoc explanations are unlikely to benefit the users of black box medical AI systems since they do not replicate but merely approximate the actual reasoning processes of these systems.^8^ As a result, critics suggest, not only do post hoc explanations fail to improve users’ understanding of black box medical AI systems, but they also fail to reduce the patient‐safety risks associated with their use and, in some cases, may even exacerbate these risks.

This article aims to defend the value of post hoc explanations against these recent critiques. Despite their limitations, we argue that post hoc explanations can still improve users’ functional understanding of black box systems, increase the accuracy of clinician‐AI teams, and assist clinicians in justifying their AI‐informed decisions, among other benefits.^9^ As a secondary aim, this article seeks to counteract what we refer to as the “silver bullet” thesis in explainable AI (XAI) research. This thesis states that the black box problem is a single, unified problem that can be solved with a single solution. We argue that the black box problem is a multifaceted one that demands varied strategies to address the many risks it presents in medicine. While post hoc explanations cannot “solve” the black box problem in medical AI, they are nevertheless a useful tool in mitigating it. Other strategies, both technical and nontechnical, will be needed to overcome or sufficiently mitigate the black box problem. But the value of post hoc explanations must not be overlooked on account of their inability to achieve these objectives in one fell swoop.

## The Black Box Problem

Many advanced AI systems—including deep neural networks, support‐vector machines, and random forests, which are some of the most popular computational architectures in machine learning at present—are black boxes. While stakeholders can scrutinize the inputs and outputs of these systems, the systems’ underlying reasoning processes are largely resistant to human understanding. AI systems can be opaque for a variety of reasons, including intentional concealment, technical illiteracy, and model size or complexity.^10^ More specifically, however, AI systems can exhibit at least two types of opacity: training opacity and inference opacity. Anders Søgaard claims that training opacity occurs “when expert humans cannot, upon inspection, say how, in general, the parameters of the DNN [deep neural networks] were induced as a result of its training data.”^11^ In contrast, inference opacity occurs “when expert humans cannot, upon inspection, say why, in general, [a model] predicts an output y as a result of an input x.”^12^

Machine‐learning systems often exhibit training opacity because learning algorithms are highly sensitive to tiny changes in the training dataset, including the order in which training data are presented to it. It is practically impossible, therefore, for users to predict the final state of the model from the data used to train it. Inference opacity arises when a model's size—specifically, the number of parameters it includes—exceeds the scope of human short‐term memory. For instance, examining a diagnostic AI system would not help a doctor understand the model's reasoning, as these models often contain millions of weighted parameters tied to specific textual or visual features that the model has learned during training.

This characteristic of AI systems gives rise to the so‐called black box problem in medicine. According to Jospeh Wadden, this problem “occurs whenever the reasons why an AI decision‐maker has arrived at its decision are not currently understandable to the patient or those involved in the patient's care because the system itself is not understandable to either of these agents.”^13^ However, the black box problem has two distinct aspects: one technical and the other ethical.

The technical aspect concerns the computational challenges that must be overcome to “open the black box” of AI. Riccardo Guidotti and coauthors identify four such challenges.^14^ First, the *model explanation* problem is that users cannot understand the underlying logic of a black box model as a whole. Unlike decision trees, for instance, support‐vector networks do not provide a chain of decision rules through which they proceed when classifying their outputs. Second, the *outcome explanation* problem is that users cannot understand the specific reasons underlying particular outputs. For example, black box systems do not provide explanations for why they classified a particular image of a skin lesion as malignant and another image as benign. Third, the *model inspection* problem is that users cannot understand specific properties of black box systems (such as the concepts or features that influence their outputs). As Guidotti and coauthors observe, researchers aim to address this problem by “providing a representation (visual or textual) for understanding some specific property of the black box model or of its predictions.”^15^ Finally, the *transparent box design* problem differs from the other three problems in that it concerns performance limitations in transparent models, rather than interpretability challenges in opaque systems. In some cases, transparent models have been found to struggle to perform as accurately as black box systems in complex tasks. To address this problem, some researchers are experimenting with various strategies that attempt to replicate the performance of black box models with straightforwardly transparent models, such as decision trees.

In contrast, the ethical aspect of the black box problem concerns the specific ethical issues and risks associated with using black box systems in medical decision‐making. Maya Krishnan identifies, for example, three such issues.^16^ According to the *justification* problem, if clinicians cannot understand the reasoning process behind an AI system's classification or recommendation, they are not justified in accepting it.^17^ According to the *antidiscrimination* problem, if clinicians cannot understand the reasoning process behind an AI system's classification or recommendation, they cannot detect whether the output's integrity has been compromised by algorithmic bias or spurious reasoning.^18^ Finally, according to the *reconciliation* problem, if clinicians cannot understand the reasoning process behind an AI system's classification or recommendation, they cannot rationally resolve disagreements between themselves and the outputs of these systems.^19^

The ethical aspects of the black box problem can be addressed without solutions to the technical aspects of the problem. For example, providing users with information about the reliability and performance characteristics of a black box system can assist them in justifying their decisions to accept or override the outputs of these systems in certain decision‐making scenarios.^20^ In the XAI literature, however, researchers tend to prioritize technical solutions, particularly post hoc explanations.

## What Are Post Hoc Explanations?

The U.S. Defense Advanced Research Projects Agency defines XAI as a research paradigm in AI that aims “to create a suite of new or modified ML [machine‐learning] techniques that produce explainable models that, when combined with effective explanation techniques, enable end users to understand, appropriately trust, and effectively manage the emerging generation of AI systems.”^21^ The most popular approaches in XAI have thus far been post hoc explanation models, which generate explanations by approximating the reasoning of a black box model on the basis of its prior record of inputs and outputs.

Post hoc explanation models come in a variety of forms.^22^ For example, post hoc explanation models can be either model specific or model agnostic. Model‐specific post hoc explanations can be applied only to particular classes of black box models (such as neural networks, support‐vector machines, and random forests). Saliency maps, for instance, are post hoc explanation models that provide visual overlays highlighting the areas of an inputted image that were most influential in determining a system's output.^23^ This type of post hoc explanation is model specific because it can be used to explain only neural networks and other gradient‐based models. In contrast, model‐agnostic approaches are those that can be applied to any type of black box model. Local interpretable model‐agnostic explanations, for instance, are post hoc explanation models that highlight visual or textual features of the input data that were most influential in determining its outputs.^24^ This type of post hoc explanation is model agnostic since it can be used to explain any class of black box model.

Post hoc explanations can also deliver global or local explanations. Global explanations attempt to explain the behavior and operations of a black box model as a whole. For example, partial dependence plots are post hoc explanations that provide a visualization of the relationship between the input and output of a black box system in a reduced feature space.^25^ Partial‐dependence plots provide global explanations because they provide insight into the relationship between specific features and predicted outcomes across the entire dataset, regardless of the values of other features. In contrast, local explanations attempt to explain the reasoning behind individual outputs. For example, Shapley additive explanations (informally called “SHAP,” whether singular or plural) offer a visualization of the contribution of each feature to the difference between the actual prediction and the average baseline prediction for individual observations within the dataset.^26^ SHAP provides local explanations because it computes the contribution of each feature to the difference between the actual prediction and the average baseline prediction for individual observations within the dataset.

## The Case against Post Hoc Explanations

Post hoc explanations have been praised for their potential to improve user understanding, to promote appropriate reliance on black box systems, and to reduce the patient‐safety risks associated with their use.^27^ Recently, however, several critics have argued that post hoc explanations cannot make good on these promises.^28^

The main reason for this is that post hoc explanations are not “genuine” or “high‐fidelity” XAI. Anantharaman Muralidharan and coauthors define high‐fidelity XAI as systems in which “the representor generated by the secondary AI emulates, to a high degree, the way the primary actually arrived at its result.”^29^ Yet, as previously discussed, post hoc explanations provide mere approximations of the actual reasoning processes taken by black box systems to arrive at their outputs. Any correspondence between a post hoc explanation and the reasoning process of a black box system is merely a matter of chance. Indeed, Cynthia Rudin goes so far as to suggest that post hoc explanations *must* be wrong: “If the explanation was completely faithful to what the original model computes, the explanation would equal the original model, and one would not need the original model in the first place, only the explanation.”^30^

As a result, critics argue, post hoc explanations cannot improve users’ understanding of black box systems or their outputs. According to Boris Babic and coauthors, for instance, post hoc explanations are a “fool's gold” that provides mere “ersatz” understanding.^31^ This is because post hoc explanation models are unable to provide *genuine* explanations for the outputs of black box systems. Rather, Babic and coauthors claim, these models are limited to offering mere rationalizations for the outputs of black box systems, and these rationalizations will, in many cases, simply appease users’ desire to critically engage with black box systems without improving their understanding of them. Such observations seem to be supported by a recent study in which post hoc explanation models were found to generate intuitively reassuring explanations for even completely untrained machine‐learning models.^32^

Other critics argue that post hoc explanations are often too shallow or uninformative to benefit users. According to Rudin, for instance, saliency maps do “not explain anything except where the network is looking.”^33^ While these explanations can identify the areas of an image that were most influential in determining a system's output, they cannot explain precisely how these areas influenced the model's reasoning, or why. Other critics highlight that explanations are not the same as justifications. For example, a post hoc explanation may explain how a model arrived at its output, but this does not demonstrate that the user ought to accept the output as true.^34^ Even if post hoc explanations could replicate the reasoning process a black box system has taken to arrive at an output, therefore, it would be insufficient to justify a user's acceptance of this output.

Still other critics argue that post hoc explanations are unlikely to promote appropriate reliance on black box systems. A range of empirical studies investigating the impact of post hoc explanations on the performance of human‐AI teams have produced concerning results. For example, Eoin M. Kenny and coauthors found that post hoc explanations do not improve users’ trust in black box systems.^35^ Malin Eiband and coauthors found that even placebic explanations (explanations that offer no insight into a recommendation or request) can promote similar levels of self‐reported trust in the outputs of black box systems compared to genuine explanations.^36^ Julius Adebayo and coauthors also found that explanations may be unable to detect spurious correlations in the reasoning processes of black box systems.^37^

Finally, critics argue that post hoc explanations may even exacerbate the very safety risks they are designed to address. Again, a variety of empirical studies investigating the impact of post hoc explanations on the performance of human‐AI teams have generated concerning findings. Automation bias, according to Kathleen Mosier and coauthors, refers to “the tendency to use automated cues as a heuristic replacement for vigilant information seeking and processing.”^38^ Mor Vered and coauthors found that post hoc explanations not only failed to reduce automation bias in users, but in some cases actually increased it.^39^ Post hoc explanations may also increase the difficulty users face when trying to detect algorithmic biases in black box medical AI systems, as post hoc explanations have been found to exhibit biases themselves.^40^ Finally, in some cases, providing post hoc explanations for the outputs of black box systems has been found to result in worse overall human‐AI team performance than simply eschewing these explanations altogether.^41^

Despite early optimism, a profound degree of pessimism concerning the potential for post hoc explanations to address the black box problem now prevails, both in medicine and beyond.^42^

## A Defense of Post Hoc Explanations

Yet post hoc explanations can benefit the users of black box systems in a variety of ways, even if they do not replicate the exact reasoning processes of these systems. Explanations from human experts likewise often do not conform to the mechanistic reasoning process through which they arrived at their judgments, yet we confidently rely on them regardless.^43^

Post hoc explanations can, for example, improve the performance of clinician‐AI teams. In a recent study, Julian Senoner and colleagues found that radiologist‐AI teams were 4.7 percentage points more accurate in identifying lung lesions from chest x‐ray images when saliency maps were used, compared to when radiologists relied on a black box system alone. More research is needed to better understand the conditions under which post hoc explanations improve clinician‐AI performance. However, these findings demonstrate that, under some conditions, post hoc explanations can have a significant positive impact on the accuracy of clinician‐AI decision‐making.

Even if post hoc explanations cannot reliably detect when a black box AI system relies on spurious correlations, as discussed previously, they can still improve users’ ability to discriminate between correct and incorrect outputs. In the aforementioned study, for instance, Senoner and colleagues found that saliency maps improved the performance of clinician‐AI teams because “domain experts supported by explainable AI were more likely to follow AI predictions when they were accurate and more likely to overrule them when they were wrong.”^44^ Post hoc explanations have also been found to reduce users’ overreliance on the outputs of AI systems in some instances.^45^ As Andrés Páez points out, moreover, “[M]odels, idealizations, simulations, and thought experiments, … play important roles in scientific understanding despite being literally false representations of their objects. In a similar vein, the methods and devices used to make black box models understandable need not be propositionally stated explanations and they need not be truthful representations of the models.”^46^

In one influential study, for example, Xinru Wang and Ming Yin found that post hoc explanations improved users’ ability to predict how the outputs of a model may change in response to changes in input data.^47^ The ability to predict a system's outputs is commonly highlighted as a key component of explainability in AI.^48^ Doctors who can predict how changes in input data will affect a system's outputs have some degree of causal understanding of the system. They are therefore better equipped to assess the weight that these outputs ought to have in their decision‐making and to explain their AI‐assisted reasoning process to patients.

Furthermore, even if post hoc explanations cannot justify a black box system's outputs,^49^ they can provide clinicians with evidence to justify their AI‐informed decisions. As Mark Theunissen and Jacob Browning have argued, post hoc explanations can be combined with “institutional explanations” to make the former more meaningful to the users of these systems.^50^ These explanations include information about “the design decisions that went into the making of the [black box] system just as much as they are the technical specifications of the system: why should we trust the company and engineers designing it, what efforts have … been made to avoid bias, and how have they worked alongside end‐users who will use these systems in their medical practice?”^51^ The justificatory power of post hoc explanations combined with institutional explanations can also be bolstered using non‐XAI methods, for instance, by providing information about the reliability of the system, the data on which the system was trained, the system's strengths and weaknesses, known “edge cases,” and so on.^52^

While critics are also correct that post hoc explanations can promote overreliance or exhibit algorithmic biases, these risks are not unique to post hoc explanations. Human explanations, too, are often biased and promote overconfidence.^53^ Despite this, experts often rely on such explanations in high‐stakes decision‐making contexts such as medicine. It is unclear why critics think we ought to hold post hoc explanations to a higher standard in this regard.

Such risks can also be mitigated. For example, biases in local explanations can be reduced through robust local training methods, such as the just train twice method.^54^ Misleading explanations can also be minimized through aggregating multiple explanations rather than providing users with a single explanation.^55^ Finally, the risk of automation bias and overconfidence can be minimized through “cognitive forcing” techniques, which Zana Buçinca and coauthors define as “interventions that are applied at the decision‐making time to disrupt heuristic reasoning and thus cause the person to engage in analytical thinking.”^56^ For example, developers could force users either to wait thirty seconds or to make their own judgments before receiving an AI system's recommendations and explanations. Developers could also incorporate the principles of “frictional AI” into the design of post hoc explanation models, which Federico Cabitza and coauthors define as “a general class of decision support aimed at achieving a useful compromise between the increase of decision effectiveness and the mitigation of cognitive risks, such as over‐reliance, automation bias and deskilling.”^57^ For instance, developers could provide users with multiple AI‐generated recommendations or explanations instead of a single output with the aim of forcing users to engage their critical faculties instead of deferring to the machine.

Post hoc explanations, therefore, can be beneficial even if they do not replicate the exact reasoning processes taken by black box systems to arrive at each of their outputs. Despite this limitation, post hoc explanations can still improve users’ functional understanding of black box systems, increase the accuracy of clinician‐AI teams, and assist clinicians in justifying their AI‐informed decisions. While post hoc explanations can promote overconfidence or produce biased explanations, these risks are also characteristic of human explanations and can be mitigated through a variety of strategies.

Post hoc explanations are not a silver‐bullet solution to the black box problem in medical AI.^58^ This is to be expected. The black box problem is not a singular problem, but a collection of distinct technical challenges and ethical risks. “Wicked” problems of this sort cannot be resolved in one fell swoop. Post hoc explanations fall short of a complete solution, yet their utility in addressing the problem must not be overlooked. Addressing the black box problem requires multiple strategies, both technical and nontechnical. Further research in the developing research area of mechanistic interpretability is promising.^59^ But a stronger focus on nontechnical strategies is also needed. AI onboarding in clinical practice must be thorough, cautious, and reversible. Clinicians must be given regular opportunities to build and develop skills in AI‐assisted decision‐making. Medical AI systems must undergo detailed postmarket surveillance of their performance and generalizability. The solution to the black box problem will not come from technological advancement alone. Skilled users and effective oversight are vital.

## Acknowledgment

The authors wish to acknowledge support from a Carlsberg Foundation Young Researcher Fellowship (CF20‐0257).
